# Associations between Surface and Rectal Temperature Profiles of Low-Birth-Weight Piglets

**DOI:** 10.3390/ani13203259

**Published:** 2023-10-19

**Authors:** Bryony S. Tucker, Kiro R. Petrovski, Jessica R. Craig, Rebecca S. Morrison, Robert J. Smits, Roy N. Kirkwood

**Affiliations:** 1 School of Animal and Veterinary Sciences, The University of Adelaide, Roseworthy, SA 5371, Australia; kiro.petrovski@adelaide.edu.au (K.R.P.); roy.kirkwood@adelaide.edu.au (R.N.K.); 2 Davies Livestock Research Centre, The University of Adelaide, Roseworthy, SA 5371, Australia; 3 Rivalea Australia Pty. Ltd., JBS Australia Pork Division, Corowa, NSW 2646, Australia; jcraig@rivalea.com.au (J.R.C.); rmorrison@rivalea.com.au (R.S.M.); 4 Research and Innovation, Australian Pork Limited, Barton, ACT 2600, Australia; robsmits@triadvice.au

**Keywords:** piglet, birth weight, temperature, rectal, surface, infrared camera, thermoregulation

## Abstract

**Simple Summary:**

Piglets experience a significant temperature drop soon after birth and the time taken to recover can impact their survival and growth. Rectal temperature is the best method currently used to monitor temperature change, however, it is invasive and requires handling which can be stressful. Infrared camera technology has improved and surface temperature has been suggested to be a good substitute for rectal temperature. A study was undertaken to investigate the utility of infrared measurements of piglets from immediately after birth to 24 h old. Results showed that surface temperature had a similar temperature pattern to rectal temperature but was highly affected by the environment and cannot be recommended as a substitute for rectal temperature measurement.

**Abstract:**

The use of infrared cameras to record surface temperature has shown some promise in older pigs, but neonatal piglets are metabolically less mature and experience rapid temperature changes during their first 24 h. The present experiment aimed to compare rectal temperature to surface temperature at the base of the ear, measured using an infrared camera, for piglets of different birth weights. During farrowing, 48 multiparous sows were monitored, and rectal and surface temperatures were recorded for their lower-birth-weight (≤1.2 kg) piglets within 3 min of birth and at 0.25, 0.50, 0.75, 1, 1.25, 1.50, 2, 3, 4, and 24 h. Piglet birth weights were assigned to one of three categories (BWC): BWC1 (≤0.80 kg), BWC2 (0.81 to 1.10 kg), or BWC3 (1.11 to 1.20 kg). Piglet rectal temperatures at 1.25 h after birth were assigned to one of three categories: RC1 (≤32.0 °C), RC2 (32.1 to 35.0 °C), or RC3 (≥35.1 °C). Surface temperatures showed a similar recovery pattern to rectal temperatures in the first 24 h across all piglet birth weights, although large and variable differences seen in the current study militate against surface temperature being an appropriate replacement for neonatal rectal temperature for use in production.

## 1. Introduction

Piglets are born wet and into an environment that is significantly cooler than conditions in utero (38–40 °C) [1,2], leading piglets to experience a dramatic reduction in body temperature during the first 15–90 min after birth [3,4,5]. The cooler ambient temperature and physical environment, combined with the presence of amniotic fluid, contribute to this decrease in temperature [6,7]. The average temperature recovery time from birth exceeds 4 h, during which it is critical for piglets to actively seek teats and consume sufficient colostrum to obtain nutrients for thermoregulation and acquire passive immunity [8,9]. In addition, failure to obtain adequate colostrum may result in hypoglycaemia and chilling, which can predispose piglets to being overlain by the sow [10].

Smaller piglets have a greater surface area to volume ratio than heavier piglets, and thus have a proportionately greater potential for heat loss and reduction in the ability to thermoregulate [4]. Consequently, heavier piglets experience a less severe postnatal drop in temperature than smaller piglets [11]. As piglets are born with little adipose tissue and no brown fat, they have a limited energy supply to maintain efficient thermoregulation [12]. As such, they rely on Muscle Non-shivering Thermogenesis to produce heat, which rapidly depletes their energy supplies [13]. Farrowing house ambient temperatures are invariably below the lower critical temperature of piglets, and so piglets must rely on the use of supplemental heat sources or interventions to assist in regulating their temperature. Previous studies have tested a variety of interventions with varying effects on temperature change and subsequent survival, such as energy supplements, drying, and warm intraperitoneal saline injections [2,14,15,16].

Rectal temperature is the gold standard for measuring the internal temperature of pigs as it is relatively unaffected by external factors. However, repetitive rectal thermometer readings to monitor temperature changes and to identify critical periods for the piglets is an invasive procedure which can become stressful for the piglet [17]. This includes removal from the crate environment and restraint, resulting in exposure to different environmental temperatures, handling stress, and potential loss of suckling time. An alternative method published recently in the literature is the use of infrared cameras for the monitoring of piglet thermal status [18,19,20]. Automatic systems generating a whole image analysis would be useful to monitor piglet thermal changes, but handheld machines are more practical and easily employed in practice. There is evidence that rectal and surface temperatures show similar diurnal trends, and that surface temperatures interact with piglet age and growth rate [19]. It has been suggested that the ocular, auricular, and nasal regions are the most commonly monitored to determine changes in heat [6]; however, many of these also require manipulation of the piglet to obtain clear images. Interestingly, the use of an aural thermometer has shown a high correlation with rectal temperature, but also requires restraint, thus offering little benefit beyond that of rectal thermometers [20].

A variety of different infrared thermometers and cameras can indicate core temperature, but are affected by factors such as distance from the animal [20]. In that study, the best targets for the infrared camera were the inner thigh and abdomen, but these too required manipulation of the piglet. The aim of the present experiment was to compare rectal temperatures with surface temperatures measured using an infrared thermal camera at the base of the ear of piglets with different body weights. We hypothesised that rectal temperature would not be highly impacted by body weight, but that surface temperature would be more sensitive to this factor.

## 2. Methods

This experiment was conducted during September and October at a commercial piggery (Rivalea, Corowa, NSW, Australia) and was approved by the Rivalea Animal Care and Ethics Committee (Protocol number 20R027) in accordance with the Australian Code for the Care and Use of Animals for Scientific Purposes (National Health and Medical Research Council, 2013).

### 2.1. Housing and Management

Multiparous Large White × Landrace sows (N = 48; mean parity 3.4 ± 1.2) were moved into their farrowing accommodations at 110 d (±2 d) of gestation, being housed in individual farrowing crates equipped with sow- and piglet-level nipple drinkers and a heat lamp positioned centrally over one side (creep area). The sows were fed 2.5 kg/day of a standard lactation diet formulated to provide 15 MJ DE/kg, 16.7% protein, and 0.90% SID lysine from entry into the farrowing house until farrowing (116 ± 1.5 d of gestation). Farrowings were observed and measurements were recorded daily from 0600 h to 1900 h. Once farrowed, sows were fed to appetite via hand feeding twice per day until weaning (21 ± 2 d of lactation). The farrowing rooms were semi-enclosed with natural ventilation and a dripper cooling system was set to automatically activate at 28 °C. Additional portable evaporative coolers were placed in the shed at sow entry and used until 2 weeks after farrowing to aid in temperature control.

### 2.2. Experimental Design

**Figure 1:**
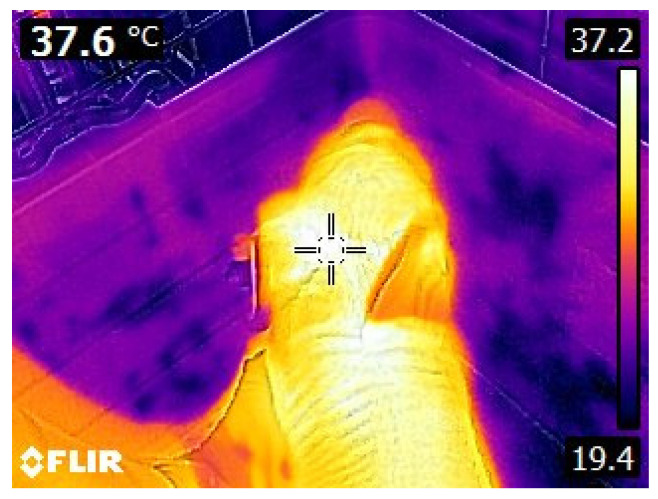
Example of a newborn piglet in the semi-enclosed container while the base-of-ear surface temperature measurement was obtained with minimal handling.

Piglets weighing ≤ 1.2 kg at birth and born during normal working hours over a 3-day farrowing period were included in this trial. Based on these constraints, 67 piglets (36 female and 31 male) born to 48 sows were included in the experiment. Piglet birth weights were assigned to one of three categories: BWC1 (≤0.80 kg), BWC2 (0.81 kg to 1.10 kg), or BWC3 (1.11 to 1.20 kg). Within 3 min of birth (0 h), piglet surface and rectal temperatures were recorded after they were moved into a semi-enclosed container positioned over a scale and were individually ear tagged, weighed, and their sex recorded. Rectal temperatures were measured using a standard digital thermometer with a lowest possible reading of 32 °C (0.3 °C sensitivity). Surface temperature was measured at the base of the right ear (Figure 1) using a FLIR E8-XT thermal camera (Teledyne FLIR, Wilsonville, OR, USA; <0.06 °C thermal sensitivity), with an emissivity value of 0.98 pre-set in the camera, held 30 cm from the piglet (using a generic ruler), and all images were saved for further confirmation [21]. Surface and rectal temperatures were recorded at 0, 0.25, 0.50, 0.75, 1, 1.25, 1.5, 2, 3, 4, and 24 h. All attempts were made to minimise further manipulation of the piglet when obtaining temperatures, though occasional placement of hand next to pig in the container was required to stabilise the animal prior to taking the photo when the piglet was wet.

### 2.3. Statistical Analysis

All statistical analyses were performed using SAS version 9.4 (Statistical Analysis Software, Cary, NC, USA). There were 737 piglet-related data point observations available for analysis. After bootstrapping the data at a root of 24 possibilities per piglet ID, 11,196 piglet observations were available for analysis. For the data analysis:

Piglet birth weights were assigned to one of three categories: BWC1 (≤0.80 kg), BWC2 (0.81 kg to 1.10 kg), or BWC3 (1.11 to 1.20 kg).

Piglet rectal temperatures at 1.25 h after birth were assigned to one of three categories: RC1 (≤32.0 °C), RC2 (32.1 °C to 35.0 °C), or RC3 (>35.0 °C).

The correlation between the rectal and surface temperatures across body weight categories were estimated using PROC CORR, with the output being the Pearson correlation coefficient (*r*) and the respective 95% confidence intervals. Correlation was deemed very high if *r* ≥ 0.90, high if *r* was between 0.70 and 0.89, moderate if *r* was between 0.50 and 0.69, low if *r* was between 0.30 and 0.49, and negligible if *r* < 0.30 [22]. The level of significance for analysis was set at *p* < 0.05.

The effects of the birth weight, time relative to birth, and the interaction of birth weight and time relative to birth on the rectal and surface temperatures of piglets were estimated using a mixed model in PROC MIXED, as presented in Equation (1)

(1) $$\begin{array}{ll} temperature= & {birth\;weight\;category}_{fr,ls,p,\;s}+{time\;related\;to\;birth}_{fr,ls,p,\;s}\\ & +({birth\;weight\;category\times time\;related\;to\;birth)}_{fr,ls,p,\;s} \end{array}$$

where *fr* = farrowing room; *ls* = litter size; *p* = sow parity; and *s* = sow. The preliminary model also tested the effects of piglet sex, but it was found to be not significant and was removed from the final model. The outputs of the model were least-square means and their respective standard errors.

## 3. Results

### Relationships between Rectal and Surface Temperatures

**Figure 2:**
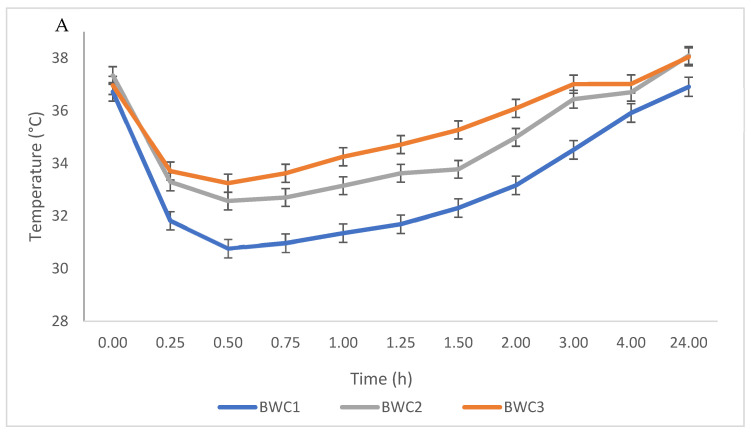

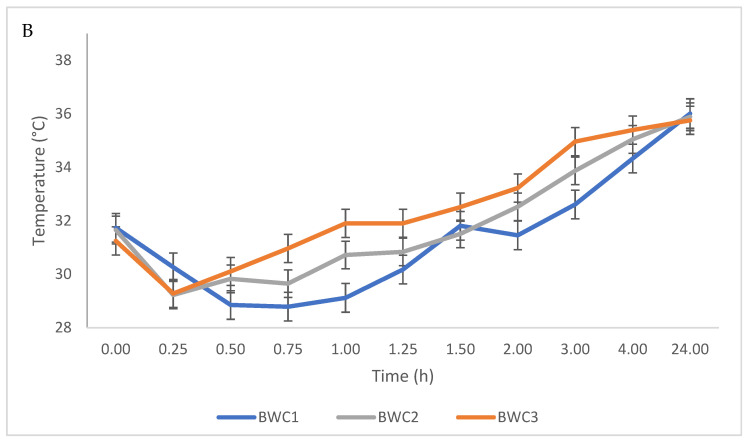
Least-square mean ± standard error for piglet rectal temperatures (**A**) and surface temperatures measured at the base of the right ear (**B**) across body weight category (BWC); adjusted for the effect of the sow, parity, litter size, and farrowing room, in 11,196 piglet observations. BWC1: ≤0.80 kg; BWC2: 0.81 kg–1.10 kg; BWC3: 1.11–1.20 kg.

Overall, rectal and surface temperatures showed a moderate correlation of 68.7% (67.7–69.7; *p* < 0.001), although the initial surface temperatures recorded were at least 4 °C lower than the rectal temperatures (Figure 2).

Rectal and surface temperatures were higher in accordance with higher body weight across time, but the surface temperature profile appeared more variable than the rectal temperature profiles (Figure 2A,B). Heavier piglets had higher rectal temperatures from 0.25 h to 24 h (*p* < 0.001; Figure 2A).

## 4. Discussion

From our data, the skin surface temperature, at the base of the ear, and rectal temperature of neonatal piglets in the postpartum period appear, at most, only moderately correlated. This is at odds with previous research (reviewed by [19]) that suggested the base of the ear is one of the best thermal windows, with a high correlation between surface and rectal temperatures. A thermal window is a skin/surface area which is well perfused by blood and, as a result, is a good “window” into the core body temperature [23]. The ear is highly vascularised and is relatively easy to access with minimal interference; as such, changes in the heat exchange can be monitored easily [3,6]. However, previous studies using infrared thermal technology in pigs have largely focused on older pigs that have thicker skin, established thermoregulation, and greater fat deposits [23]. Small piglets have little natural insulation, a higher surface area to volume ratio, and limited energy reserves to assist in thermoregulation, compared to their heavier-born counterparts [1,12]. Furthermore, piglets are born wet, moving from a regulated temperature in utero of approximately 39 °C to a significantly cooler external environment [24]. Therefore, previously validated methods of measuring surface temperature using infrared thermal technology for older pigs are not as applicable to neonatal piglets.

Birth weight significantly influenced the rectal and surface temperature profiles of piglets during their first 24 h of life. None of the smallest piglets (BWC1) had rectal temperatures above 35 °C at 1.25 h. However, if they did have a relatively higher temperature, <35 °C, their rate of survival increased. A relationship between temperature (and hypothermia) and birth weight has been established for piglet preweaning survival [1]. That the observed effects might have been different if the temperatures were measured at a different time cannot be discounted, but we rationalised that by 1.25 h, any prenatal influence of the sow should have worn off as the piglet would have dried and sucked. Further, by 1.25 h, thermoregulation should be improved, and piglets would be on the upwards curve of temperature recovery, as previously documented [4,5].

Similar to previous research, piglet temperatures had decreased by 0.25 h post-birth, although the start of recovery was evident from 0.5 h rather than 1 h as previously documented [4]. This was followed by a gradual increase until 24 h. The difference in recovery patterns between our study and that of Caldara et al. [4] presumably reflects differences in the farrowing environments, including, but not limited to, ambient temperatures, heat management, and piglet drying [4]. In our study, the difference between the rectal and surface temperature recovery curves highlights the influence that external factors have on surface temperature. It is known that smaller piglets have a greater surface area to volume ratio, and therefore presumably lose heat more rapidly and would be affected more quickly by environmental changes.

These findings, along with the physiology of the piglet, highlight the importance of good management from early life to ensure optimal survival. Providing an optimal environment from birth is critical to support this; however, arguably the most important strategy to improve survival is to ensure fast and adequate access to colostrum, particularly for low-birth-weight piglets. Colostrum intake is positively related to rectal temperature and provides the critical energy required to thermoregulate and build an immune response, thus setting the piglets up for survival [25,26]. Being able to monitor this temperature change using technology would be a valuable tool for piglet management and welfare.

Other studies which have focused on the use of infrared thermal technology in piglets have concluded that this technology has the potential to be an effective, less invasive tool for temperature measurement, albeit not identical to rectal temperature [18]. There are many factors affecting the surface temperature using infrared technology, such as the angle of the camera, background lighting, ambient temperature, humidity, and stress to the piglet [27]. One major challenge of recording surface temperature is the manipulation of the piglet required to obtain an accurate reading. The most effective method is to pick up the piglet and manoeuvre it so that exact and repeatable measures can be recorded. However, in doing so, the piglet is exposed to different environmental conditions within the crate which may trigger a stress response, which has been shown to have a substantial impact on thermal window temperature readings, in addition to using energy from their limited supply [27]. These challenges, and our results, suggest that the use of a handheld infrared camera is impractical for direct application by producers. However, with increasing interest in machine learning and automation in pig production, there is potential for further research and application in this area [28].

## 5. Conclusions

Surface temperature measured using infrared thermal camera technology at the base of the ear produces large variations compared to rectal temperature, although the recovery pattern was similar. Therefore, these devices cannot be recommended for use in commercial settings at this stage. Birth weight and temperature are directly related and should be considered together when monitoring piglets.

## Data Availability

The data presented in this study are available on reasonable request from the corresponding author.

## References

[1] Kammersgaard T.S., Pedersen L.J., Jørgensen E. (2011). Hypothermia in neonatal piglets: Interactions and causes of individual differences. J. Anim. Sci..

[2] Andersen H.M., Pedersen L.J. (2016). Effect of radiant heat at the birth site in farrowing crates on hypothermia and behaviour in neonatal piglets. Animal.

[3] Villanueva-Garcíaa D., Mota-Rojasb D., Martínez-Burnesc J., Mora-Medinae P., Salmerónf C., Gómezb J., Boscatob L., Gutiérrez-Pérezf O., Cruzf V., Reyesb B. (2021). Hypothermia in newly born piglets: Mechanisms of thermoregulation and pathophysiology of death. J. Anim. Behav. Biometerology.

[4] Caldara F.R., Dos Santos L.S., Machado S.T., Moi M., de Alencar Naas I., Foppa L., Garcia R.G., de Kassia Silva Dos Santos R. (2014). Piglets’ surface temperature change at different weights at birth. Asian-Australas. J. Anim. Sci..

[5] Vande Pol K.D., Cooper N., Tolosa A., Ellis M., Shull C.M., Brown K., Alencar S. (2020). Effect of method of drying piglets at birth on rectal temperature over the first 24 hours after birth. Transl. Anim. Sci..

[6] Gómez-Prado J., Pereira A.M.F., Wang D., Villanueva-García D., Domínguez-Oliva A., Mora-Medina P., Hernández-Avalos I., Martínez-Burnes J., Casas-Alvarado A., Olmos-Hernández A. (2022). Thermoregulation mechanisms and perspectives for validating thermal windows in pigs with hypothermia and hyperthermia: An overview. Front. Vet. Sci..

[7] Bienboire-Frosini C., Muns R., Marcet-Rius M., Gazzano A., Villanueva-García D., Martínez-Burnes J., Domínguez-Oliva A., Lezama-García K., Casas-Alvarado A., Mota-Rojas D. (2023). Vitality in Newborn Farm Animals: Adverse Factors, Physiological Responses, Pharmacological Therapies, and Physical Methods to Increase Neonate Vigor. Animals.

[8] Rooke J.A., Bland I.M. (2002). The acquisition of passive immunity in the new-born piglet. Livest. Prod. Sci..

[9] Theil P.K., Lauridsen C., Quesnel H. (2014). Neonatal piglet survival: Impact of sow nutrition around parturition on fetal glycogen deposition and production and composition of colostrum and transient milk. Animal.

[10] Pandolfi F., Edwards S.A., Robert F., Kyriazakis I. (2017). Risk factors associated with the different categories of piglet perinatal mortality in French farms. Prev. Vet. Med..

[11] Cooper N., Vande Pol K.D., Ellis M., Xiong Y., Gates R. (2019). Effect of piglet birth weight and drying on post-natal changes in rectal temperature. J. Anim. Sci..

[12] Herpin P., Damon M., Le Dividich J. (2002). Development of thermoregulation and neonatal survival in pigs. Livest. Prod. Sci..

[13] Bienboire-Frosini C., Wang D., Marcet-Rius M., Villanueva-García D., Gazzano A., Domínguez-Oliva A., Olmos-Hernández A., Hernández-Ávalos I., Lezama-García K., Verduzco-Mendoza A. (2023). The Role of Brown Adipose Tissue and Energy Metabolism in Mammalian Thermoregulation during the Perinatal Period. Animals.

[14] Declerck I., Dewulf J., Decaluwé R., Maes D. (2016). Effects of energy supplementation to neonatal (very) low birth weight piglets on mortality, weaning weight, daily weight gain and colostrum intake. Livest. Sci..

[15] Vande Pol K., Tolosa A., Shull C., Brown C., Alencar S., Ellis M. (2021). Effect of drying and warming piglets at birth on pre-weaning mortality. Transl. Anim. Sci..

[16] Tucker B.S., Petrovski K.R., Kirkwood R.N. (2022). Neonatal piglet temperature changes: Effect of intraperitoneal warm saline injection. Animals.

[17] Gimsa U., Brückmann R., Tuchscherer A., Tuchscherer M., Kanitz E. (2022). Early-life maternal deprivation affects the mother-offspring relationship in domestic pigs, as well as the neuroendocrine development and coping behavior of piglets. Front. Behav. Neurosci..

[18] Kammersgaard T.S., Malmkvist J., Pedersen L.J. (2013). Infrared thermography--a non-invasive tool to evaluate thermal status of neonatal pigs based on surface temperature. Animal.

[19] Zakari F.O., Akefe I.O., Uchendu C. (2021). Comparison of diurnal rectal and body surface temperatures in large white piglets during the hot-dry season in a tropical Guinea savannah. J. Therm. Biol..

[20] Schmid S.M., Büscher W., Steinhoff-Wagner J. (2021). Suitability of different thermometers for measuring body core and skin temperatures in suckling piglets. Animals.

[21] Soerensen D., Clausen S., Mercer J., Pedersen L. (2014). Determining the emissivity of pig skin for accurate infrared thermography. Comput. Electron. Agric..

[22] Hue D.T., Williams J.L., Petrovski K., Bottema C.D.K. (2021). Predicting colostrum and calf blood components based on refractometry. J. Dairy Res..

[23] Soerensen D.D., Pedersen L.J. (2015). Infrared skin temperature measurements for monitoring health in pigs: A review. Acta Vet. Scand..

[24] Muns R., Nuntapaitoon M., Tummaruk P. (2016). Non-infectious causes of pre-weaning mortality in piglets. Livest. Sci..

[25] Decaluwé R., Maes D., Wuyts B., Cools A., Piepers S., Janssens G.P.J. (2014). Piglets’ colostrum intake associates with daily weight gain and survival until weaning. Livest. Sci..

[26] Devillers N., Le Dividich J., Prunier A. (2011). Influence of colostrum intake on piglet survival and immunity. Animal.

[27] Magnani D., Gatto M., Cafazzo S., Stelletta C., Morgante M., Nanni Costa L. Difference of surface body temperature in piglets due to the backtest and environmental condition. Proceedings of the Animal Hygiene and Sustainable Livestock Production; the XVth International Congress of the International Scociety for Animal Hygiene.

[28] Lu M., He J., Chen C., Okinda C., Shen M., Liu L., Yao W., Norton T., Berckmans D. (2018). An automatic ear base temperature extraction method for top view piglet thermal image. Comput. Electron. Agric..

